# Porous Micropillar Arrays with Oil Infusion: Fabrication, Characterisation, and Wettability Analysis

**DOI:** 10.3390/mi16121419

**Published:** 2025-12-17

**Authors:** David Gibbon, Prabuddha De Saram, Azeez Bakare, Navid Kashaninejad

**Affiliations:** 1 School of Engineering and Built Environment, Griffith University, 170 Kessels Road, Nathan, Brisbane, QLD 4111, Australia; 2 Queensland Quantum and Advanced Technologies Research Institute, Griffith University, 170 Kessels Road, Nathan, Brisbane, QLD 4111, Australia; 3 School of Environment and Science, Griffith University, 170 Kessels Road, Nathan, Brisbane, QLD 4111, Australia

**Keywords:** micropillars, modified wettability, porosity, superhydrophobic, superoleophilic, self-cleaning surfaces

## Abstract

Superhydrophobic micropillar surfaces, inspired by the lotus leaf, have been extensively studied over the past two decades for their self-cleaning, anti-friction, anti-icing, and anti-corrosion properties. In this study, we introduce a simple and effective method for introducing porosity into polydimethylsiloxane (PDMS) micropillar arrays using salt templating. We then evaluate the wetting behaviour of these surfaces before and after infusion with perfluoropolyether (PFPE) oil. Apparent contact angle and sliding angle were measured relative to a non-porous control surface. Across five porous variants, the contact angle decreased by approximately 5° (from 157° to 152° on average), while the sliding angle increased by about 3.5° (from 16.5° to 20° on average). Following PFPE infusion, the porous arrays exhibited reduced sliding angles while maintaining superhydrophobicity. These results indicate that introducing porosity slightly reduces water repellency and droplet mobility, whereas PFPE infusion restores mobility while preserving high water repellency. The change in wettability following PFPE infusion highlights the potential of these surfaces to function as robust, self-cleaning materials.

## 1. Introduction

Surfaces engineered for specific wetting behaviours play an important role across biomedical devices, microfluidic systems, aviation and marine technologies, owing to their diverse functional properties [1,2]. Beyond simple hydrophobic/hydrophilic behaviour, micro/nanotopographies and chemistry can produce superhydrophobic or superhydrophilic responses and, crucially, tune contact angle hysteresis (CAH) to control droplet mobility [3,4,5,6].

Micropillar arrays form a widely studied class of such engineered surfaces [7,8]. With appropriate geometry (pitch, height, diameter), they routinely deliver high apparent contact angles (CA) and water repellency using accessible, repeatable fabrication techniques such as soft lithography and rapid prototyping. Geometry governs wetting characteristics, i.e., by controlling the solid-area fraction, one can achieve superhydrophobicity and tune CAH and pinning behaviour through hierarchical micropillar structures. Larger pillars can be more easily reproduced and are effective in achieving superhydrophobic surfaces; however, these surfaces usually experience much higher CAH due to the accentuation of the pinning effect. Smaller micropillars have been used to not only achieve superhydrophobic surfaces but also omniphobic surfaces. Advanced micropillar structures, such as re-entrant structures, can achieve tuneable wettability properties through additional parameters, including the properties of the cap and dual material structures.

Among available materials for fabricating such pillars, polydimethylsiloxane (PDMS) is widely preferred in biomedical and lab-on-a-chip systems because it is chemically inert, biocompatible, optically clear, elastomeric, and straightforward to replicate by soft lithography from a diverse range of moulds (PMMA, silicon, printed resins, etc.) [4,9,10,11,12]. PDMS being inherently hydrophobic, the ability to achieve superhydrophobicity without extremely low solid area fractions makes it further suitable for micropillar arrays fabrication for wettability engineering.

Porous substrates have attracted increasing interest for microfluidic applications due to their enhanced surface area, tuneable wetting properties, and fluid absorption capabilities. Recent advances in porous materials such as silica, silicon, and hydrogels have opened new avenues for microfluidic design [13,14,15]. More recently, there have been developments in porous polymers for use within microfluidics [14]. These porous polymers offer extreme wetting contrasts and large liquid uptake, with absorption often far exceeding their own mass. Porous PDMS has been shown in many studies to be flexible, durable and superhydrophobic [2,16,17,18,19,20,21,22,23,24]. Several fabrication strategies for introducing porosity into polymer substrates have been reported. Among them, sacrificial templating or emulsion casting are widely accepted methods in porous polymer fabrication. For example, porous PDMS can be created through mixing a dissolvable additive, such as NaCl or sugar, with uncured PDMS and dissolving it out with water once cured [17,19]. Other additives, such as simple water or solvent emulsions, can create the desired porosity after drying/boiling [20,22,23,25].

Porous PDMS has shown promising applications in the biomedical engineering space. Lo et al. showed an improved, lightweight, stretchable and lower impedance ECG/EMG sensor created from coated PDMS sponge, using the sugar templating process [26]. Porous PDMS has also been used to drastically increase gas absorption (10×) in microfluidic devices, allowing more precise control of conditions within microfluidic devices [2]. In another study, porous PDMS has been used as a slow-release reservoir for drug dispersion within a microfluidic system, allowing controlled release and completely passive drug dispersion. Thin-layer porous PDMS has been used to model the blood–brain barrier on a chip [23].

A promising strategy in surface engineering is to combine microtexture with porosity to create porous micropillars. These structures behave as hierarchical surfaces, where the micropillars form the primary level of texture and the internal pores provide a secondary, finer hierarchy that enhances liquid–solid interactions. Previous studies have explored this concept in various materials. For instance, Zheng et al. fabricated silicon micropillars using electrochemical etching [15]. This process was effective in producing a layer of porous sponge-like silicon. However, the limitations of this method were that the sponge-like porosity could not be introduced uniformly throughout the entire micropillar or surface due to the electromagnetic distribution on the surface varying with the features. Agonafer et al. also utilised porous micropillar structures with a single pore for the retention of low-surface-tension fluids through capillarity [14]. They demonstrated that porous micropillars retain lubricants more effectively than their non-porous counterparts. The potential applications of porous PDMS micropillars are very promising, with micropillars already being utilised for cell implantation, as the increased surface area of the porous structure could provide more secure attachment points for cells. The interconnected porosity could also provide pathways for gas exchange, the delivery of drugs, or for modelling membranes, as has been shown in porous PDMS research.

Compared to conventional hierarchical structures, porous microstructures act as capillary reservoirs for lubricants, retaining low-surface-tension oils more effectively than nonporous counterparts and stabilising liquid-infused states under shear or repeated use. This underpins the self-cleaning behaviour of liquid-infused surfaces (LIS). At the same time, static contact angles can be pushed into the superhydrophobic region by controlling micropillar parameters, and surface porosity characteristics can influence the sliding angle. This enables droplet mobilisation with minimal effort, shedding particulates and residues on the surface. The infused liquid forms a liquid interface that can deter the attachment of biofoulants, making these surfaces attractive for anti-fouling biomedical and environmental applications.

Although porous micropillar surfaces have been investigated using various materials and fabrication methods, most prior studies have focused either on achieving porosity or tailoring micropillar geometry, rather than systematically combining both to understand their coupled influence on wettability and droplet mobility. Approaches such as electro-chemical etching and single-pore incorporation often produce non-uniform porosity or limited control over pore size and distribution, restricting insight into how hierarchical micro–nano features govern liquid–solid interactions. Moreover, while PDMS has been used in soft-lithography-based porous microfabrication, reports on fully porous PDMS micropillar arrays that maintain structural fidelity and controllable pore characteristics remain scarce. While porous silicon micropillars and bulk or membrane-format porous PDMS structures (foams and films) have been reported, we found no studies demonstrating fully interconnected porous PDMS micropillar arrays fabricated via a soft-lithography–compatible templating route and characterised for wettability and drop-let mobility. In this work, we address this gap by fabricating PDMS micropillar arrays with tuneable through-pillar porosity using a simple, reproducible templating method. By systematically varying porosity parameters, we elucidate how pore structure and pillar geometry collectively affect surface wettability, contact angle hysteresis, and sliding behaviour, thereby establishing design principles for hierarchical PDMS surfaces with potential self-cleaning and anti-fouling functionality.

## 2. Materials and Methods

### 2.1. Materials

Sylgard-184 PDMS base and curing agent (Dow, Midland, MI, USA) were mixed at a 10:1 (*w*/*w*) ratio. Analytical-grade sodium chloride (>99%, particle size < 75 μm after sieving) was used as the porogen. Milli-Q water (18.2 MΩ·cm) and isopropanol (IPA, ≥99.5%) were used throughout the fabrication and cleaning steps. A portable dehumidifier (Airrex ADH1000, Rex Nordic Group, Askola, Finland) maintained the relative humidity (RH) between 45% and 56% during powder preparation.

### 2.2. Fabrication of Micropillar Moulds

Micropillar master moulds were fabricated by CO_2_ laser ablation of 4.5 mm-thick PMMA sheets (Trotec Speedy 300, 10.6 μm wavelength, Trotec Laser GmbH, Marchtrenk, Austria). A 9 W average laser power and a scan speed of 1 inch·s^−1^ were used with a single pass. The lens focal length was 2 inches, and the scanning resolution was set to 1000 pulses per inch (PPI).

### 2.3. Preparation of Porous PDMS Micropillars

PDMS prepolymer and curing agent were mixed at a 10:1 ratio and combined with NaCl powder at five mass fractions, as summarised in Table 1. The mixture was stirred for 5 min until it became homogeneous and then poured onto the laser-ablated PMMA moulds to form ~1.5 mm-thick substrates. The samples were placed under vacuum at 0.7 Torr for 5 min to ensure full penetration into the micropillar cavities and removal of trapped air. The cast substrates were cured at room temperature for 12 h, followed by post-curing at 80 °C for 4 h, and allowed to cool to room temperature.

### 2.4. Salt Leaching and Drying

The cured PDMS/salt composites were immersed in Milli-Q water containing 5 vol % IPA and placed in an ultrasonic bath for 2 days (total ultrasonic time 2 h), during which the leaching solution was replaced four times. After leaching, the samples were subjected to a 30 min IPA exchange and dried in an oven at 60 °C for 8 h.

### 2.5. Liquid Infusion

Vacuum-assisted infusion was used to introduce oil into the micropores. Krytox GPL 105 (perfluoropolyether, PFPE) (Chemours Company, Wilmington, DE, USA) was selected for its weak interactions with PDMS relative to hydrocarbon or silicone oils, its extremely low vapour pressure, its high chemical stability, and its omniphobic characteristics. For the infusion process, the micropillar substrates were fully submerged in PFPE oil and subjected to two vacuum–release cycles. After air bubbles were no longer observed during vacuuming, excess oil trapped between the micropillars was gently removed by blowing air across the surface. The substrates were then held vertically to allow remaining excess oil to drain under gravity, with a gentle air stream applied to accelerate the removal process.

**Figure 1:**
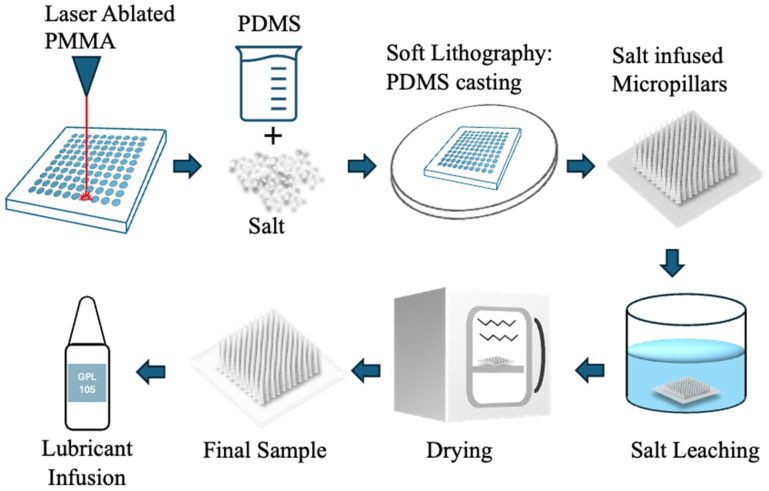
Schematic of the fabrication process for porous PDMS micropillars. The process begins with CO_2_ laser ablation of a PMMA substrate to generate the micropillar mould. PDMS prepolymer is mixed with NaCl particles and cast onto the laser-ablated mould. After curing, the PDMS/salt composite is demoulded to produce salt-infused micropillars. The embedded salt is removed by aqueous leaching, and the samples are subsequently dried to obtain porous PDMS micropillar arrays. The final structures can then be infused with Krytox GPL 105 oil for the fabrication of liquid-infused porous micropillar surfaces.

An overview of the complete fabrication workflow, from laser ablation to salt leaching and drying, is summarised in Figure 1.

### 2.6. Surface Characterisation

Scanning electron microscopy (SEM) was used to examine pore morphology and to determine the size distribution of surface pores. SEM imaging was conducted on an Apreo 2s system (Thermo Fisher Scientific, Waltham, MA, USA) operated in low-vacuum mode (30 Pa, 5–20 kV accelerating voltage, ~10 mm working distance). Imaging parameters were adjusted depending on the required magnification. For geometrical measurements of the microstructures, we used a laser scanning microscope (Olympus LEXT OLS5100, Olympus Corporation, Tokyo, Japan) equipped with a 10× objective lens.

Pore morphology and size distribution were quantified from low-vacuum SEM micrographs using a custom Python script (Python 3.14, NumPy, Pillow). Images were background-corrected using Gaussian blur subtraction, normalised by quantile or histogram matching, and segmented using either Otsu’s thresholding or median + k × MAD thresholding. The fraction of pixels above the selected threshold was used to estimate the surface-porosity ratio.

### 2.7. Wettability Characterisation

Static contact angles (CA) and sliding angles (SA) were measured using a ThetaFlex optical tensiometer (Biolin Scientific, Gothenburg, Sweden). A 10 µL Milli-Q water droplet was dispensed at 20 µL s^−1^ onto five distinct regions of each sample, parallel to the pillar axis. For SA, the stage was tilted from 0° to 55° at 90° per min. The average of five measurements was reported for each sample, and the standard deviation is given as ± error.

## 3. Results

### 3.1. Geometrical Characterisation

Laser scanning micrographs were used to measure the basic dimensions. The pillars exhibited a shark-fin-like profile in the height maps, which arises from multiple overlapping laser passes during the CO_2_ ablation process, consistent with observations reported in previous studies [10]. The average height of the pillars from base to tip is 717 µm, which is comparatively large, with most in the literature ranging from 5 µm on the small end up to about 350 µm on the larger end [11]. Despite their larger size, the fabricated arrays displayed good uniformity in pillar geometry, making them suitable for reliable comparative analysis.

The laser-ablated triangular pillars also showed intrinsic surface roughness in the 1–10 μm range, generated by the CO_2_ laser process. This hierarchical roughness may contribute to a rose-petal-like wetting response and increase droplet pinning, which is consistent with the higher sliding angles observed for these structures.

**Figure 2:**
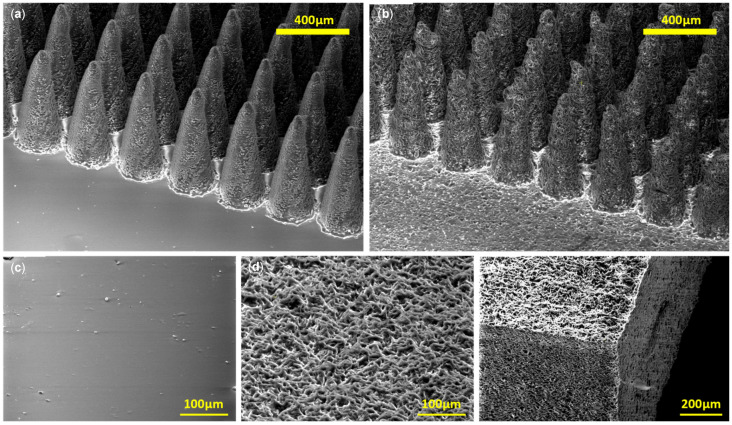
Scanning electron microscope images of non-porous and porous PDMS structures. (**a**) Non-porous micropillars with laser-machined PMMA mould. (**b**) Porous micropillars with the same mould as (**a**). (**c**) Plain PDMS surface moulded from unmachined parts of the PMMA mould. (**d**,**e**) Porous PDMS plain surface and sliced porous PDMS substrate. Interconnected pores are clearly visible on the sliced sample.

Figure 2 presents SEM images of both non-porous and porous PDMS structures. Figure 2a shows the non-porous micropillars, fabricated using the laser-machined PMMA mould. Figure 2b shows porous micropillars replicated from the same mould, while Figure 2c illustrates a plain PDMS surface from an unmachined region of the mould. Figure 2d,e display porous plain surfaces and sliced porous PDMS substrates, clearly showing interconnected pore networks.

**Figure 3:**
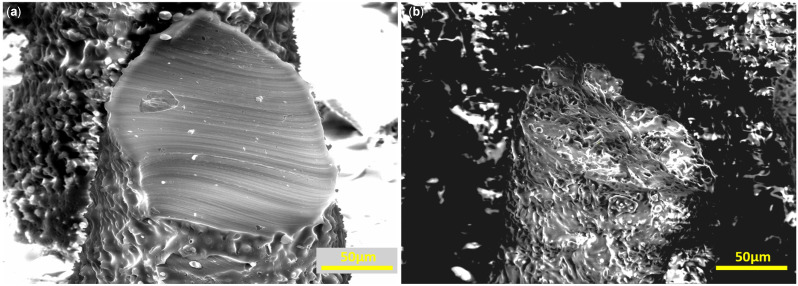
Magnified images of sliced micropillars. (**a**) Nonporous micropillar (**b**) Porous micropillar with 2:1 salt to PDMS *w*/*w* ratio. Interconnected porosity throughout the pillar surface and the pillar core is visible.

The porosity observed in Figure 2b,d,e corresponds to the 2:1 NaCl-to-PDMS formulation (Sample 5 in Table 1, 66.7 wt% salt). This sample exhibited a noticeable volume reduction after cleaning and drying, shrinking to 86.3% of its original length (equivalent to 64.3% of its original volume), indicating significant structural collapse. The cross-sectional SEM images confirm that the porosity fully penetrated the substrate, with no undissolved salt remaining, as shown in Figure 3b. All samples returned to a weight approximately equal to that of the PDMS component, confirming near-complete removal of the salt. As also noted in porous PDMS foam studies [16], all salt-containing samples expanded considerably during leaching. Higher salt fractions corresponded to greater swelling during the cleaning process.

Figure 3 shows magnified images of sliced micropillars. Figure 3a depicts a non-porous pillar, while Figure 3b shows a porous pillar from Sample 5 (2:1 ratio, see Table 1). The porous pillar exhibits substantial micron-scale roughness and interconnected porosity through both the surface and core. The lower porosity seen in the pillar cross-section, compared with the plain surface in Figure 2d, is likely due to limited infiltration of the salt particles into the pillar cavities during casting.

### 3.2. Characterisation of Surface Porosity on Plain Surfaces

**Figure 4:**
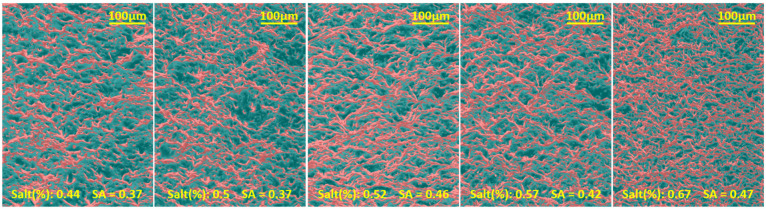
Coloured SEM images of plain surfaces based on pixel intensity. Red colour overlay depicts brighter areas. The brightness threshold was selected by manually inspecting the images and identifying clear, solid surface areas. All the images were normalised, histogram matched, and the same threshold was used.

To isolate the effect of porosity alone, plain (non-pillared) PDMS surfaces were fabricated using the same salt-templating procedure. SEM micrographs of these surfaces were acquired at identical magnifications to enable direct comparison. Surface porosity was then quantified from the SEM images by analysing the darker regions, which correspond to pores, using a custom Python workflow. The intensity of each pixel was measured, and image statistics were used to calculate the solid-area fraction for each salt concentration. Figure 4 shows the colour-mapped SEM images used for this analysis.

Overall, the solid-area fraction increased with salt content; however, the analysis also indicates that the density of smaller pores rises with increasing salt percentage. This behaviour may be due to the accumulation of fine salt particles near the bottom of the mould, which can obstruct larger particles from reaching the surface, thereby increasing the apparent solid-area fraction.

### 3.3. Surface Wettability Characterisation

Porosity alone, even without micropillars, increases the contact angle of PDMS. Plain PDMS surfaces fabricated with the above ratio exhibit a contact angle of 116.4 ± 0.1°, whereas flat porous surfaces with salt fractions of 0.44, 0.50, 0.52, 0.57, and 0.67 show contact angles of 123.5 ± 0.0°, 125.6 ± 0.2°, 125.6 ± 0.4°, 123.1 ± 0.2°, and 125.6 ± 0.4°, respectively. These measured values are lower than those predicted by the Cassie–Baxter equation using the corresponding solid area fractions, suggesting that the surfaces do not maintain a fully suspended Cassie state. Instead, the results indicate a mixed Cassie–Baxter/Wenzel regime, where partial liquid penetration into the porous structure reduces the apparent contact angle relative to the ideal Cassie prediction.

**Figure 5:**
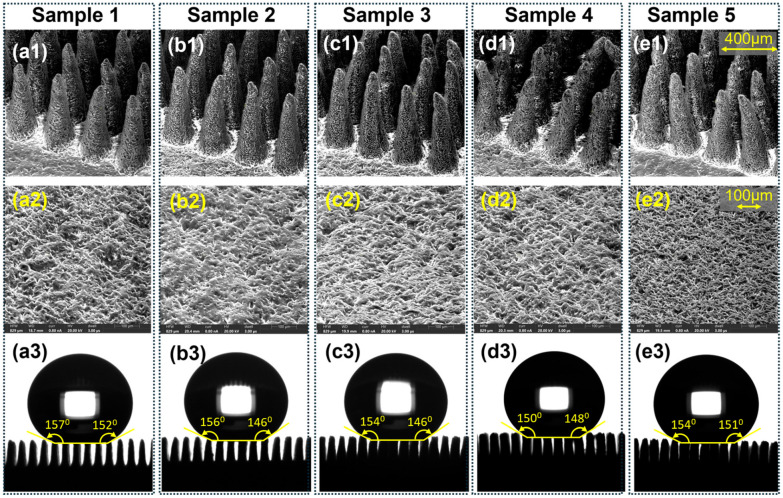
Morphology, surface porosity, and droplet profiles of porous PDMS micropillar arrays fabricated with increasing NaCl content (Samples 1–5). (**a1**–**e1**) Cross-sectional SEM images of the micropillar arrays, showing the progressive increase in pore density and surface roughness with higher salt fractions (scale bar: 400 μm). (**a2**–**e2**) Top-view SEM micrographs of the corresponding porous PDMS surfaces, illustrating the evolution of pore size and distribution (scale bar: 100 μm). (**a3**–**e3**) Apparent contact angle images of 10 μL water droplets on each sample, confirming the preservation of Cassie–Baxter wetting across all porosity levels.

With the introduction of micropillars, a 10 μL water droplet rested on approximately six pillars in the Cassie–Baxter state, resulting in an apparent contact angle of 157° ± 2.6°, placing the surface in the superhydrophobic regime. Figure 5 shows the micropillar morphology, surface porosity, and corresponding droplet profiles for Samples 1–5. It confirms the Cassie–Baxter wetting state prior to quantitative wettability analysis.

The sliding angle was 17° ± 3.6°, slightly higher than typical superhydrophobic surfaces (usually <10°). This increased sliding angle is attributed to the large pillar size and the microscale surface roughness introduced by the CO_2_ laser ablation, both of which enhance droplet pinning. The conical pillar geometry also causes the droplet to spread along the vertical axis, further increasing pinning. Contact angle hysteresis was similarly elevated at 25.4° ± 7.9°, which is substantial for a micropillar surface, and was measured from the difference between the measured advancing (AD) and receding (RE) contact angles. Despite this, the fabricated structures demonstrated excellent wetting resistance, comparable to that of more complex microfabrication approaches.

#### Wettability of Porous Micropillars

**Figure 6:**
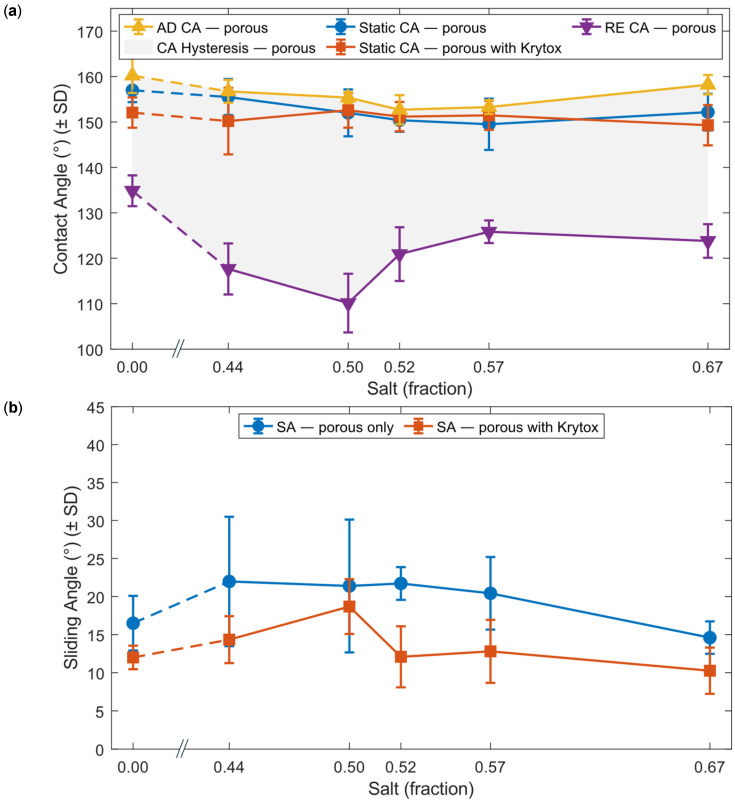
Wettability measurements of porous and oil-infused porous substrates. (**a**) Contact angle-related parameters of porous micro-pillared sample and porous with PFPE oil-infused samples. (**b**) Sliding angle (SA) of a 10 µL droplet on porous micropillars and the same substrates with oil infused. In both (**a**,**b**), the salt fraction of 0 represents the control micropillar surfaces without porosity. In both graphs, the x-axis is clipped between 0 and 0.44. Error bars represent ± one standard deviation for each data point.

Porous micropillars also exhibit superhydrophobic Cassie–Baxter state wetting for 10 μL droplets. However, the contact angle is slightly less than that of the non-porous pillars and shows some variation with the changing porosity. Figure 6 illustrates the influence of salt content on contact angle and sliding angle.

Comparison of the solid-area fraction (Section 3.2) with the contact angle data reveals a complex relationship with increasing salt content. Although higher salt percentages do not necessarily reduce the solid-area fraction, they increase the number of pores while decreasing their size, which effectively reduces the characteristic feature size of the upper roughness in the hierarchical surface.

For the droplet, the effective solid-area fraction arises from both the micropillar geometry and the underlying porous network, with the micropillars contributing most significantly. As expected, the non-porous control array increased the native PDMS contact angle from ~115° to ~157°, while the presence of porosity produced only minor reductions in the apparent contact angle but consistently increased the sliding angle.

This behaviour can be attributed to a mixed Cassie–Baxter/Wenzel wetting state, in which micropores beneath the droplet become locally wetted (Wenzel), while the droplet as a whole remains in Cassie–Baxter contact with the larger pillars. This is similar to the rose-petal effect, where surfaces can be hydrophobic but still strongly adhesive.

For the highest salt fraction (0.57–0.67), the apparent contact angle increased slightly, and the sliding angle decreased. This likely results from the formation of smaller pores in the 67 wt% sample (Sample 5), which may not fully wet at the pillar tips, permitting a partial Cassie–Baxter state on the upper structures and reducing pinning.

### 3.4. Oil Infusion on Porous Structures

**Figure 7:**
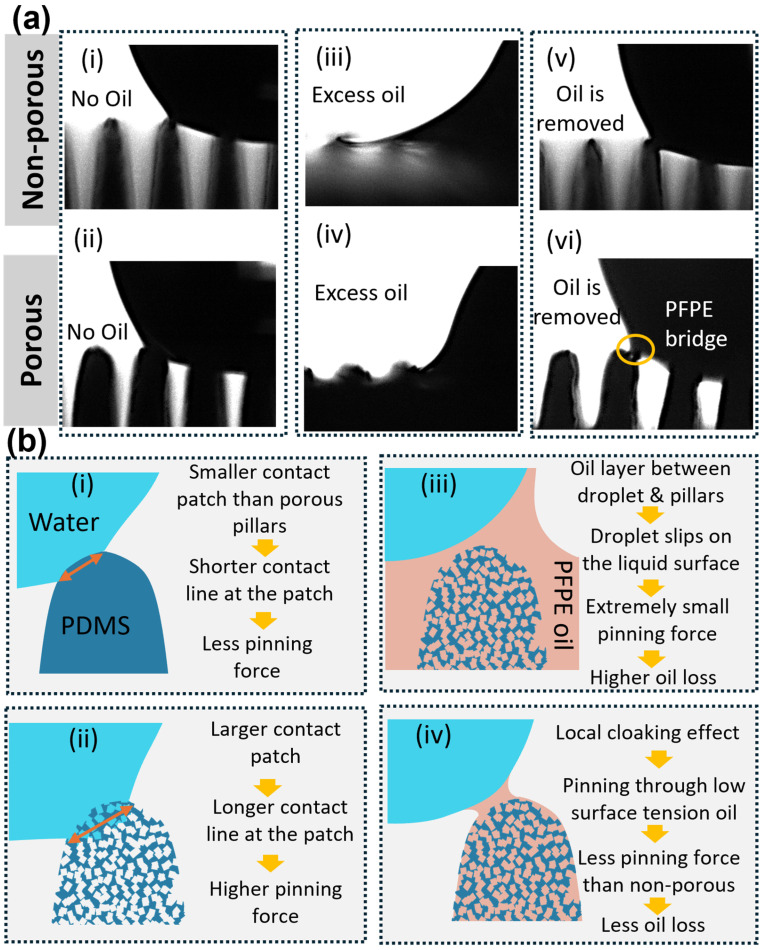
Depinning dynamics of water droplets on non-porous and porous PDMS micropillars under different PFPE oil-infusion conditions. (**a**) Side-view images showing droplet depinning on non-porous (**i**,**iii**,**v**) and porous (**ii**,**iv**,**vi**) pillars, with no oil (**i**,**ii**), with excess PFPE oil (**iii**,**iv**), and after removal of excess oil (**v**,**vi**). Surfaces containing excess PFPE oil exhibit smooth and continuous recession of the contact line, whereas non-infused and oil-removed surfaces show intermittent depinning. A PFPE bridge is visible in panel (**vi**) as shown by the yellow circle, indicating partial oil transfer during droplet motion. (**b**) Schematic interpretation of the corresponding depinning behaviour of a droplet on (**i**) non-porous pillars, (**ii**) porous pillars, (**iii**) porous pillars with excess PFPE oil, and (**iv**) porous pillars with excess PFPE removed, illustrating differences in contact-patch size (orange double arrow), pinning strength, formation of cloaking layers, and the effects of oil retention or loss on droplet mobility.

Wettability was further examined after infusing the porous micropillars with PFPE oil. In experiments before removing excess oil, care was taken to ensure the pillars were not completely submerged in the oil layer, allowing the pillar tops to remain exposed. Water droplets exhibited clear cloaking by PFPE oil, and the water–PDMS contact line was not well defined. The sliding angle for all samples decreased markedly to approximately 3° or less, indicating the formation of a stable lubricating oil film beneath the droplet when excess oil is present. The density of Krytox GPL 105 (1.94 g/mL), higher than that of water, supports the presence of this stable underlying film (Figure 7a(iii,iv)).

As the cloaking effect prevents contact between water and the solid surface, the remaining excess PFPE oil was removed, leaving the infused oil in the pores. This creates a surface that shows Cassie–Baxter wetting on a large scale while a droplet contacts micro-oil pockets on micropillar tops. This partially flattens the differences in porosity across the micropillars. As we can see in Figure 6a, PFPE oil-infused samples show a minimum variation in contact angle compared to plain porous samples.

More importantly, oil infusion significantly reduced the sliding angle across all porous samples by more than 5° relative to the non-infused state. This is attributed to the inability of water to penetrate the microstructures once oil occupies the pore spaces. The thin lubricating oil layer reduces solid–liquid contact and produces a local cloaking effect (Figure 7a(vi),b(iv)).

Droplet depinning occurs when the depinning force exceeds the pinning force associated with surface tension. Because the micropillars lack sharp edges, the pinning patch can shift to lower-energy locations. Porous pillars exhibit larger contact patches than non-porous ones (Figure 7a(i,ii)), explaining their initially higher sliding angles. After oil infusion, however, oil-filled pores substantially decrease pinning forces due to the lower surface tension of Krytox GPL 105 relative to water, producing the reduced sliding angles seen in Figure 6b.

This reduction in pinning is a major advantage of oil infusion, enabling potential applications in self-cleaning and anti-biofouling surfaces. Low pinning forces allow droplets to easily slide off, removing debris from the surface. For biofouling, cells and proteins encounter a largely liquid interface, decreasing their likelihood of attachment. The chemical inertness and low surface affinity of PFPE oils further enhance resistance to fouling.

Despite the favourable wetting performance, long-term lubricant retention remains a limitation. As illustrated in Figure 7a(vi), PFPE oil can be partially withdrawn into the droplet during depinning events. Long-term studies are therefore needed to evaluate the durability of the infused oil layer.

## 4. Conclusions

In this study, we established a simple and reproducible salt-templating method for fabricating porous PDMS micropillar arrays. The approach produced previously unreported PDMS structures with interconnected porosity extending through both the substrate and the pillars. Morphological analysis confirmed uniform pillar geometry and complete removal of the sacrificial salt. Wettability measurements showed that introducing porosity caused a slight reduction in apparent contact angle and an increase in sliding angle, primarily due to enhanced droplet pinning similar to the rose-petal effect.

In addition to modifying surface energy and adhesion, the interconnected porous structure offers potential for storing and controllably releasing substances (such as drugs or alternative oils) and for increasing effective surface area in absorption- or reaction-based applications, whereas the oil-infused configuration is better suited to functionalities that require reduced contact-line pinning and enhanced droplet mobility. Following PFPE oil infusion, the porous micropillars exhibited sliding angles lower than those of the original non-porous pillars, even though the apparent contact angle remained largely unchanged. This demonstrates the ability of oil infusion to significantly reduce surface stickiness and promote droplet mobility.

These findings highlight the potential of oil-infused porous micropillar arrays as self-cleaning and biofouling-resistant surfaces. The presence of liquid pockets and the reduced solid-area fraction can minimise the attachment of external particles and biological contaminants. Future work should quantitatively assess long-term oil retention and directly evaluate anti-biofouling performance to verify the durability and functional stability of these surfaces.

## Figures and Tables

**Table 1:** Composition of porous PDMS samples.

Sample	NaCl (g)	PDMS (g)	Ratio	*w*/*w* %
1	1.6	2	0.8:1	44.4%
2	2	2	1:1	50%
3	2.2	2	1.1:1	52.4%
4	2.6	2	1.3:1	56.5%
5	4	2	2:1	66.7%

## Data Availability

The data that support the findings of this study are available from the corresponding author upon reasonable request.
